# Development of latent Interferon alpha 2b as a safe therapeutic for treatment of Hepatitis C virus infection

**DOI:** 10.1038/s41598-019-47074-y

**Published:** 2019-07-26

**Authors:** Iram Gull, Muhammad Shahbaz Aslam, Imran Tipu, Roohi Mushtaq, Tehseen Zamir Ali, Muhammad Amin Athar

**Affiliations:** 1 Institute of Biochemistry and Biotechnology, Quaid-i-Azam Campus, University of the Punjab, Lahore, 54590 Pakistan; 2 Punjab University Health Centre, Quaid-i-Azam Campus, University of the Punjab, Lahore, 54590 Pakistan

**Keywords:** Genetic engineering, Protein design

## Abstract

Interferon therapy for the treatment of hepatitis C virus infection has very limited clinical application due to short serum half-life and side effects of therapy in systemic route of administration. In the present study, we have focused to improve the interferon therapy by overcoming the limitation of side effects. We hypothesized that latent interferon alpha 2b (IFNα2b) produced by fusion of Latency associated protein (LAP) domain of TGFβ and IFNα2b having HCV NS3 protease cleavage site as linker that will be activated only at target site (liver) by viral protease (HCV NS3 protease) present on the surface of infected cells. The fusion proteins were expressed in *pichia pastoris* as homodimer and cleaved by recombinant HCV NS3 protease *in vitro* into two fragments corresponding to the IFNα-2b and LAP respectively. The latency of chimeric proteins and biological activity after treatment with HCV NS3 protease was assessed by cytopathic effect inhibition assay in A594 cells infected with encephalomyocarditis virus (EMCV) and reduction in HCV viral load in Huh7 cells. The HCV NS3 protease was present on the surface of HCV replicating Huh7 cells in amount that activated half of the effective concentration (EC_50_) of latent IFNα2b fusion protein. As free circulating HCV NS3 protease was not detected in sera from chronic HCV patients and *in vitro* cleavage of intact latent IFNα2b fusion protein was not observed with peripheral blood mononuclear cells (PBMCs) isolated from chronic HCV patients, thus there are less likely chances of activation and off target binding of latent IFNα2b to show side effects during systemic route of administration. Therefore, most of the side effects of interferon can be overwhelmed at the cost of 50% reduced biological activity. Thus, the use of latent IFNα2b can be considered again as an option for treatment of HCV infection in combination with direct acting antivirals rather than alone with improved safety profile.

## Introduction

Cytokines are small (<30 kDa) signaling proteins that mediate cell-cell interaction and modulate cellular activity in infection, inflammation and malignancy^1^. Food and Drug Administration (FDA) has approved some of these cytokines for therapeutic purposes. However, the use of these potent recombinant cytokines is limited due to adverse side effects. These side effects are induced by administration of cytokines at high dose to achieve therapeutic concentration in tissue being targeted^2^.

Hepatitis C virus (HCV) is known as major cause of chronic liver diseases and reported to infect ~170 million people worldwide. The persistent infection of HCV leads to chronic hepatitis, liver cirrhosis and hepatocellular carcinoma^3^. The standard of care therapy for HCV infection was pegylated interferon with ribavirin^4^. Together with therapeutic benefits, interferon alpha preparations are associated with diverse nature of adverse effects like fatigue, flu like symptoms, neuropsychiatric (depression, cognitive dysfunction, mania), autoimmune effects (systemic lupus erythematosus, immune mediated hemolysis), ischemic effects (pericarditis, sick sinus syndrome producing arrhymias), interstitial pneumonitis, hematologic side effects (pernicious anemia, neutropenia), renal complications (acute nephrotic syndrome, interstatial nephritis) and dermatologic reactions of interferon injections (erythema, vasulitis, necrosis)^5–20^.

Underlying cause of each adverse effect of interferon alpha is the short serum half-life, pleiotropic nature, presence of its receptors on multiple cells and capacity to release multiple cytokines^21^. The adverse effects of interferon therapy lead to number of patients to discontinue the treatment^10^. Having been used for many years after FDA approval, interferon alpha preparations (interferon alpha 2b, peginterferon alpha 2a, peginterferon alpha 2b) have now carry black box warnings^1^.

Currently, hepatitis C therapy is focused on use of direct acting antivirals (DAA) with interferon free regimes. However, these antiviral drugs are also facing challenges i-e. high cost issues, HCV resistance in treatment failure, and treatment of special population (patients with liver cirrhosis)^22,23^. The antiviral therapy should be efficient, well tolerated, affordable and easy in access to care.

The strategies used to overcome the side effects of therapeutic drugs or proteins include: targeting them at disease site or use them as pro-drug (latent form). The approach of targeted delivery gives benefits in terms of localized therapeutic action of biomolecules with reduced dose, retain efficacy while avoiding non-specific binding in non-target cells^24–27^. The approaches utilized for targeted delivery of interferon to liver includes: fusion of interferon alpha 2 with antibody specific to liver restricted asialoglycoprotein receptor^28^ and galactosyl modified interferon specific in binding with hepatocyte specific asialoglycoprotein receptor^29^.

Second approach to overcome the side effects of systemic delivery of cytokines is to engineer them in latent form that becomes active only at target site by enzymatic cleavage^30^. The therapeutic proteins have been made latent either by: (i) fusion of N-terminal to its C-terminal through linkers^31,32^, (ii) by fusion of inhibitory peptide^33^ and (iii) by fusion of latency associated protein of TGFβ^30^. The linkers reported for conditional activation of latent cytokines at pathological sites or in specific targeted tissues include: matrix metalloprotease (MMP), human immunodeficiency virus (HIV) protease and HCV NS3 protease cleavage sites. Previously, for eradication of HCV infected cells, HCV NS3 protease activated recombinant toxin named “zymoxin” was delivered to the HCV infected cells that was made latent by fusion of antitoxin^33^.

In the present study, to improve the pharmacokinetics and to overcome the pleiotropic effects of systemic delivery of interferon, interferon alpha 2b was engineered to make it latent by providing the protective shell of latency associated protein of TGFβ fused at either N or C-terminal via HCV NS3 protease cleavage site as linker. Here, we hypothesized that latent interferon alpha 2b will be activated by the HCV NS3 protease present at the surface of HCV infected hepatocytes as Sakata *et al*.^34^ has reported the presence of HCV NS3 protease on the surface of HCV infected hepatocytes.

## Results

### Structural design of latent interferon alpha 2

**Figure 1:**
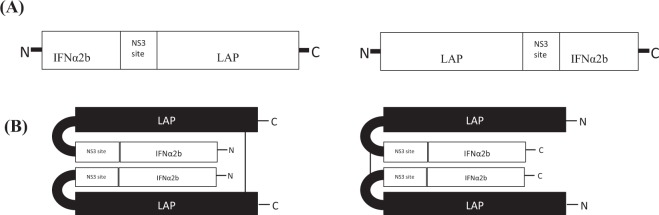
Design of latent interferon alpha 2b fused with LAP domain of human TGFβ via HCV NS3 protease cleavage site as linker. (**A**) Linear sequence of IFNα2b fused with LAP in both conformations. (**B**) Putative folding of fusion proteins showing formation of intermolecular disulfide bridge between cysteine residue 224 and 226 to confer latency to IFNα2b.

The latent IFNα-2b was developed in two conformations; i) fusion of LAP at N-terminus of IFNα-2b, and ii) fusion at C-terminus of IFNα-2b. In both conformations, HCV NS3 protease cleavage site was introduced as liker between fusion partners through SOE-PCR (Fig. 1A). In the protein sequence of human TGFβ, LAP domain is comprised of amino acids from 30–278 while amino acids from residue 279–390 constitute active TGFβ part. In the present study, we fused LAP fragment with the IFNα-2b either at N or C-terminus. The cysteine residues at position 224 and 226 are involved in intermolecular disulfide bridge formation to confer latency to the fused IFNα-2b (Fig. 1B).

### Construction of recombinant expression plasmids

**Figure 2:**
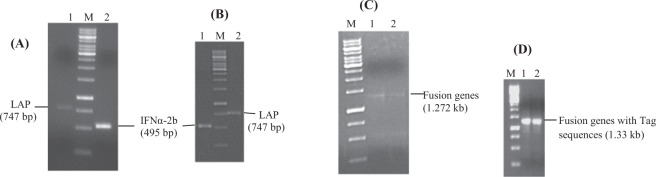
Construction of full length fusion genes IFNα2b-NS3-LAP and LAP-NS3-IFNα2b through SOE-PCR and OPW-PCR. (**A**) Amplification of LAP (Lane 1) and IFNα2b (Lane 2) gene with FP1/RP1 and FP2/RP2 primers respectively for fusion gene IFNα2b-NS3-LAP. (**B**) Amplification of IFNα2b (Lane 1) and LAP (Lane 2) gene with FP1’/RP1’ and FP2’/RP2’ primers respectively for fusion gene LAP-NS3-IFNα2b. (**C**) Amplification of fusion gene IFNα2b-NS3-LAP (Lane 1) and LAP-NS3-IFNα2b (Lane 2) with insertion of HCV NS3 protease site between fusion partners through SOE-PCR. (**D**) Appendage of Tag sequence at 5′ end of fusion gene IFNα2b-NS3-LAP (Lane 1) and LAP-NS3-IFNα2b (Lane 2) through OPW-PCR. Lane “M” represents the DNA marker.

**Figure 3:**
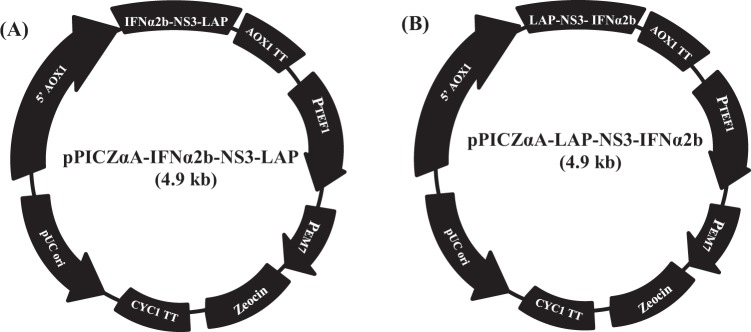
Construction of recombinant expression plasmids. (**A**) pPICZαA-IFNα2b-NS3-LAP and (**B**) pPICZαA-LAP-NS3-IFNα2b. The fusion genes were ligated downstream to the yeast α-factor secretion signal peptide sequence under the control of alcohol oxidase (AOX1) promoter.

The gene sequence of IFNα2b (495 bp) was fused to LAP gene sequence (747 bp) via HCV NS3 protease cleavage site (30 bp) as linker in both conformations through SOE-PCR (Fig. 2C). The tag peptide sequence (Kex2-Histidine tag- Gly/Ser spacer-Enterokinase site) was introduced at 5′ end of each full length fusion gene through OPW-PCR and full length fusion genes (IFNα2b-NS3-LAP and LAP-NS3-IFNα2b) of size 1.33 kb were successfully amplified (Fig. 2D). The primers introduced *xbaI* and *xhoI* sites at 5′ end and 3′ end of each fusion gene. Each full length fusion gene was ligated with pPICZαA vector restricted with *xbaI* and *xhoI* restriction enzymes. The recombinant expression plasmids were denoted as pPICZαA-IFNα2b-NS3-LAP and pPICZαA-LAP-NS3-IFNα2b. In recombinant expression plasmids, gene were present under the control of alcohol oxidase (AOX1) promoter followed by α-factor secretion signal of *Saccharomyces cerevisiae* that direct the protein export to medium (Fig. 3).

In frame cloning and sequence of full length fusion genes were confirmed by sequencing (data not shown).

### Transformation and screening of transformants

**Figure 4:**
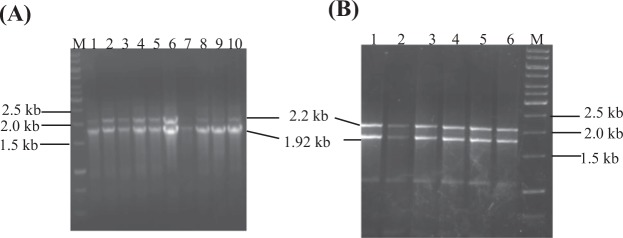
Verification of pPICZαA-IFNα2b-NS3-LAP and pPICZαA-LAP-NS3-IFNα2b expression cassette in GS115 genome by colony PCR and PCR using genomic DNA of integrants with primers 5′ AOX1 and 3′AOX1. (**A**) Analysis of integrants by colony PCR. Lane M: DNA marker, Lane 1–5: colony PCR products of pPICZαA-IFNα2b-NS3-LAP integrants, Lane 6–10: colony PCR products of pPICZαA-LAP-NS3-IFNα2b integrantion in GS115 genome. (**B**) Analysis of fusion gene integration using genomic DNA of integrants. Lane 1–3: amplicons using gemonic DNA of integrants with AOX1 primers. Lane M: DNA marker, Lane 1–3: amplicon of pPICZαA-IFNα2b-NS3-LAP, Lane 4–6: amplicons of pPICZαA-LAP-NS3-IFNα2b integration in GS115 genome. The band of size 1.9 kb represents fusion gene flanked by 588 bp of AOX1 sequence whereas 2.2 kb band showed amplification of AOX1 gene (indicating Mut^+^ phenotype of integrants).

The recombinant vectors were linearized with *SacI* enzyme and transformed in GS115 cells of *pichia pastoris*. The clonal transformants were replicated on YPD agar plates containing different concentrations of zeocin. Five putative transformants showing growth on YPD plates having 1500 µg/ml zeocin, were randomly selected. The integration of expression cassette in *pichia pastoris* genome was verified by colony PCR and by PCR using genomic DNA of selected transformants with AOX1 primers. The amplification of two bands of size 2.2 kb and 1.92 kb indicated that the integrants were of Mut^+^ phenotype. The band of 2.2 kb corresponds to the amplification of alcohol oxidase (AOX1) gene while band of 1.92 kb is the result of amplification of fusion gene (1.33 kb flanked by 588 bp of AOX1 sequence (Fig. 4A,B).

### Expression of fusion genes in Pichia pastoris

**Figure 5:**
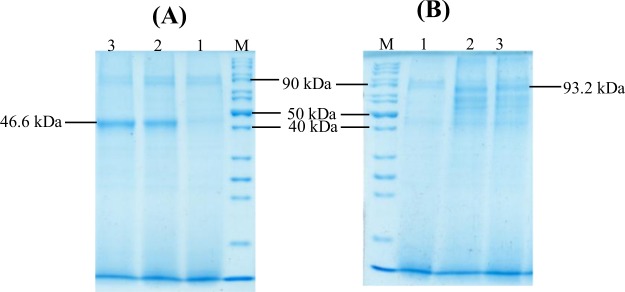
Expression analysis of fusion genes under reducing and non-reducing SDS-PAGE. (**A**) SDS-PAGE analysis of expression under reducing conditions and (**B**) under non-reducing conditions. Lane M: protein size marker, Lane 1: Total secretory proteins of GS115 transformed with pPICZαA plasmid, Lane 2: total secretory proteins of GS115 expressing IFNα2b-NS3-LAP fusion protein, Lane 3: total secretory proteins of GS115 showing expression of LAP-NS3-IFNα2b fusion protein. The bands of size 46.6 kDa indicate monomeric form of fusion proteins and 93.2 kDa bands indicate homodimeric form of fusion proteins.

The positive colonies were cultured and induced with 1.0% methanol (after every 24 hours) to express recombinant fusion proteins for 120 hours at 20 °C in BMMY pH 5.0 medium with shaking at 300 rpm. The total secretory protein from induced culture supernatant were precipitated and analyzed by SDS-PAGE under both reducing and non-reducing conditions. Under non-reducing conditions, a band of ~93.2 kDa showed the formation of homodimer of each fusion protein while this size reduced to ~46.6 kDa in gel under reducing conditions. The ~46.6 kDa band corresponds to the estimated size of monomeric fusion protein (Fig. 5A,B).

### Purification and characterization of fusion proteins

**Figure 6:**
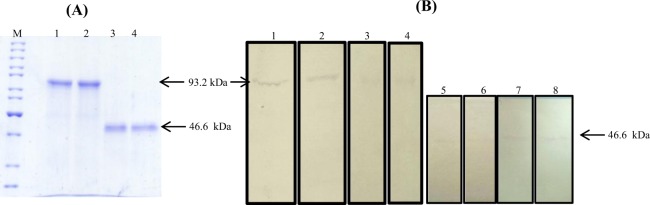
SDS-PAGE analysis and western blotting of purified fusion proteins under reducing and non-reducing conditions. (**A**) SDS-PAGE analysis of purified fusion proteins. Lane M: Protein size marker, Lane 1 & 2: purified IFNα2b-NS3-LAP and LAP-NS3-IFNα2b fusion proteins analyzed under non-reducing conditions (93.2 kDa band indicated by arrow). Lane 3 & 4: purified IFNα2b-NS3-LAP and LAP-NS3-IFNα2b fusion proteins respectively analyzed under reducing conditions (46.6 kDa band indicated by arrow). (**B**) Western blot analysis of purified proteins. Homodimers of IFNα2b-NS3-LAP and LAP-NS3-IFNα2b detected by mouse anti-human IFNα2 antibodies (blot 1 & 2) and by mouse anti-human LAP antibodies (blot 3 & 4). IFNα2b-NS3-LAP and LAP-NS3-IFNα2b detected with mouse anti-human IFNα2 antibodies (blot 5 & 6) and by mouse anti-human LAP antibodies (blot 7 & 8) by western blot under reducing conditions. (The resultant image of western blots is a product of time-averaged data). The full-length blots are presented in Supplementary Figs 1–3.

The fusion proteins secreted into the supernatant after induction of cultures were purified by affinity chromatography using Ni-NTA resin under native conditions through His-tag present at N-terminus of both fusion protein. The proteins were pure after one step purification as indicated by the presence of single band on non-reducing (~93.2 kDa) and reducing (46.6 kDa) SDS-PAGE gel (Fig. 6A). The average yield of fusion proteins was 15–17 mg/L. The purified proteins were treated with enterokinase to remove His-tag from proteins. The proteins were purified using purification columns of cut off, MCO 30 kDa. The removal of His-tag from purified proteins was confirmed by western blotting using anti- histidine antibodies. The western blot analysis performed using both mouse anti-human IFNα2 antibody and mouse anti-human LAP antibodies (Fig. 6B) confirmed that fusion proteins were comprised of both fusion partners (IFNα2b and LAP).

### *In vitro* cleavage of fusion proteins by HCV NS3 protease

**Figure 7:**
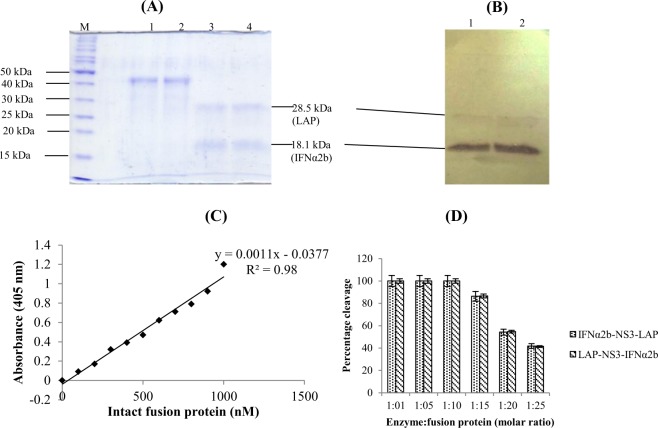
*In vitro* cleavage of intact fusion proteins by HCV NS3 protease. (**A**) SDS-PAGE analysis of *in vitro* cleavage by NS3 protease. Lane M: Protein size marker, Lane 1 & 2: Intact fusion proteins IFNα2b-NS3-LAP and LAP-NS3-IFNα2b respectively. Lane 3 & 4: Cleavage of intact fusion proteins IFNα2b-NS3-LAP and LAP-NS3-IFNα2b respectively by recombinant HCV NS3 protease (in 10:1 molar ratio). Two bands of size 28.5 kDa and 18.1 kDa indicating the LAP fragment and IFNα2b fragment respectively after cleavage of intact fusion proteins (43.5 kDa). (**B**) Western blot analysis of IFNα2b and LAP fragment produced after cleavage of fusion proteins IFNα2b-NS3-LAP (Lane 1) and LAP-NS3-IFNα2b (Lane 2) by HCV NS3 protease using mouse anti-human IFNα2 antibodies and mouse anti-LAP antibodies as primary antibodies. (**C**) Standard curve of intact fusion protein plotted against absorbance versus intact fusion protein concentration prepared after sandwich ELISA. (**D**) Analysis of *in vitro* cleavage of fusion proteins at different molar ratio of enzyme: fusion protein by sandwich ELISA. Complete cleavage was observed in 1:10 NS3 protease to substrate molar ratio under reaction conditions.

The intact fusion proteins were completely cleaved in to two fragments of size 28.5 kDa (LAP fragment) and 18.1 kDa (IFNα2b fragment) in 1: 10 molar ratio of HCV NS3 protease and fusion proteins (Fig. 7A). The *in vitro* cleavage of fusion proteins was also monitored by recording the absorbance at 405 nm after sandwich ELISA using standard curve of fusion proteins as reference (Fig. 7C). The maximum absorbance was recorded in negative control (without addition of NS3 protease). The decrease in absorbance was directly proportional to the cleavage of fusion proteins whereas no signal was recorded in absence of intact fusion protein indicating complete cleavage. In the case of *in vitro* cleavage assay of fusion protein by HCV NS3 protease at different molar ratios, no signal was recorded at 405 nm. It confirmed the complete cleavage of proteins by protease (Fig. 7D). The detection of two fragments of IFNα2b and LAP using mouse anti-human IFNα2 antibodies and mouse anti-LAP antibodies as primary antibodies in western blotting further confirmed the cleavage of fusion proteins (Fig. 7B).

### *In vitro* cleavage of fusion proteins by sera and peripheral blood mononuclear cells (PBMCs) of CHC (chronic HCV) patients

To investigate the possibility of activation of latent fusion proteins by systemic delivery of to HCV patients, the *in vitro* cleavage assay was also performed by using the sera and PBMCs of HCV patients with different viral loads. The percentage cleavage was analyzed by sandwich ELISA. The cleavage of any fusion protein was neither observed with sera nor with PBMCs of chronic HCV genotype 3a patients (Table 1). The result may lead to the conclusion that either HCV NS3 protease is not present in sera of chronic HCV patients or NS3 protease activity was inhibited by some factors in serum. To find the answer, we used the SensoLyte^®^ 520 HCV NS3 Protease Assay Kit “Fluorimetric” (AnaSpec) to detect the presence of HCV NS3 protease in serum of CHC patients at different stages of liver fibrosis. The HCV NS3 protease was not detected in any serum sample of CHC patients (Table 1). As the cleavage of fusion proteins was also not detected by PBMCs of CHC patients, it eliminated the possibility of latent intact fusion protein activation in blood during systemic route of delivery.

**Table 1:** Analysis of various parameters of CHC patient at different stages of liver injury and *in vitro* cleavage of fusion proteins by sera and PBMCs of CHC patients.

Stage of liver fibrosis	No. of patients	Gender (Male/Female)	Genotype (1a/3a)	Viral load	*In vitro* cleavage of fusion proteins by serum	*In vitro* cleavage of fusion proteins by PBMCs	NS3 protease level in sera of CHC patients
F0	27	16/11	2/18	1.639 × 10^5^ ± 1.94 × 10^4^	Not detected	Not detected	Not detected
F1	21	14/7	2/11	5.95 × 10^5^ ± 1.23 × 10^4^	Not detected	Not detected	Not detected
F2	24	15/9	2/14	4.85 × 10^6^ ± 4.38 × 10^5^	Not detected	Not detected	Not detected
F3	29	20/9	8/18	1.21 × 10^8^ ± 2.70 × 10^7^	Not detected	Not detected	Not detected
F4	17	10/7	4/11	4.16 × 10^6^ ± 1.29 × 10^6^	Not detected	Not detected	Not detected

### *In vitro* cytopathic effect inhibition assay

The biological activity of latent and *in vitro* activated fusion proteins was assessed by inhibition in cytopathic effect induced by EMC virus in human lung carcinoma cell line A594. The results showed that latent form of fusion proteins had negligible antiviral activity (0.16% and 0.2% for LAP-NS3-IFNα2b and IFNα2b-NS3-LAP) as compared to the standard IFNα2b (EC_50_ 8.0 pg/ml). The *in vitro* activated latent fusion proteins IFNα2b-NS3-LAP and LAP-NS3-IFNα2b showed 64% (EC_50_ of 22.22 pg/ml) to 66% (EC_50_ of 23.52 pg/ml) reduced biological activity respectively than standard IFNα2b (10 & 15% reduced biological activity respectively on molar basis). The effect of culture media on activation of NS3 protease was checked by activation of latent fusion proteins during cytopathic effect inhibition assay. The results showed that biological activity of real time activated fusion proteins was further reduced to 4% i-e. EC_50_ of 25 pg/ml and 26.66 pg/ml was recorded for IFNα2b-NS3-LAP and LAP-NS3-IFNα2b respectively (Table 2).

**Table 2:** Comparative antiviral activity of IFNα2b fusion proteins.

Fusion protein	EC_50_ (pg/ml)	IU/ml	Specific activity (U/mg)	Percentage activity relative to IFNα2b
Latent IFNα2b-NS3-LAP	4000	2.1 × 10^7^	5.20 × 10^5^	0.2%
Pre-activated IFNα2b-NS3-LAP	22.22	1.3 × 10^7^	9.36 × 10^7^	36%
Real time activated IFNα2b-NS3-LAP	25	2.0 × 10^7^	8.32 × 10^7^	32%
Latent LAP-NS3-IFNα2b	5000	2.7 × 10^7^	4.16 × 10^5^	0.16%
Pre-activated LAP-NS3-IFNα2b	23.52	1.4 × 10^7^	8.84 × 10^7^	34%
Real time activated LAP-NS3-IFNα2b	26.66	1.3 × 10^7^	1.3 × 10^7^	30%
IFNα2b (standard)	8.0		2.6 × 10^8^	100%

### *In vitro* antiviral activity against HCV genotype 3a

**Figure 8:**
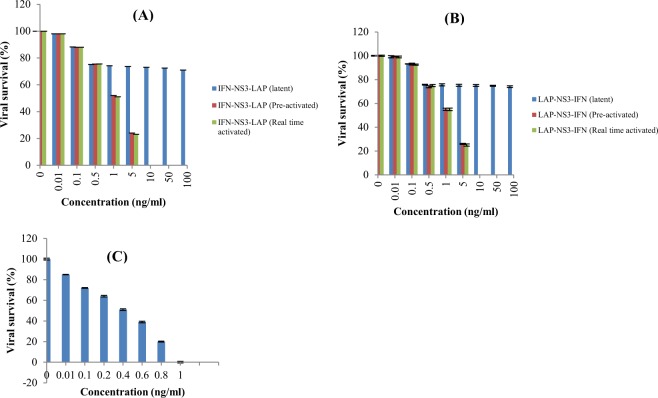
Anti-HCV activity of IFNα2b and chimeric proteins. The percentage reduction in HCV titer by latent and activated: (**A**) IFNα2b-NS3-LAP fusion proteins; (**B**) LAP-NS3-IFNα2b fusion proteins and (**C**) unconjugated recombinant IFNα2b. The latent fusion proteins showed approximately 25% reduction in viral load at the concentration of 0.5 ng/ml and maximum 29% (for IFNα2b-NS3-LAP) and 26% (for LAP-NS3-IFNα2b) reduction in HCV viral titer at 100 ng/ml. Pre-activated and real time activated fusion protein showed 50% reduction in viral titer at concentration of ~1.0 ng/ml. The standard IFNα2b showed EC_50_ against HCV at 0.4 ng/ml.

The antiviral activity of IFNα2b fusion proteins was also assessed by measuring reduction of HCV genotype 3a viral load replicating in liver cell line Huh7 culture using fusion proteins at different concentrations. The results showed that latent fusion proteins in both conformations also had antiviral activity in dose dependent manner. The viral load was reduced at same rate by latent and pre-activated forms of IFNα2b fusion protein up to the concentration of 0.5 ng/ml (Fig. 8A,B). The reduction in viral load was 25% at this concentration. The latent forms showed negligible reduction in viral load at further higher concentrations. The pre-activated fusion proteins showed 50% reduction in viral load at concentration of ~1.0 ng/ml. For fusion proteins activated during assay, the 50% reduction in viral load was recorded at the same concentrations as for pre-activated fusion proteins (Fig. 8A,B). The recombinant IFNα2b (PBL Biomedical Laboratories, Piscataway, NJ) was used as positive control. The 50% reduction in viral load for positive control was recorded at 0.4 ng/ml (Fig. 8C). The EC_50_ of unconjugated and activated form of IFNα2b fusion proteins are same on molar basis. The results revealed the activation of half of the EC_50_ of latent IFNα2b fusion protein by HCV NS3 protease present on the surface of HCV replicating Huh7 cells.

## Methods

### Use of human blood/serum

HCV infected patient serum samples were obtained with informed consents and all experiments with blood/serum were performed in accordance with relevant guidelines and regulations, following the approval of the institutional ethical committee, University of the Punjab, Lahore, Pakistan.

### Design of fusion proteins

To develop latent interferon alpha 2b (IFNα2b) fusion protein, the latency associated protein (LAP) domain of human TGFβ has been fused in two conformations: i) LAP was fused either at N-terminus or ii) at C- terminus of IFNα2b. At splicing junction of each fusion gene, HCV NS3 protease cleavage site (EDVVCCSMSY) was introduced as cleavable linker. The fusion proteins were designated as LAP-NS3- IFN and IFN-NS3-LAP. The N- terminus of each fusion gene was tagged with peptide sequence “N-Kex2 site-His tag- Gly/Ser spacer-enterokinase site-C” to facilitate processing of protein in *pichia pastoris* expression system, protein purification and removal of His-tag from protein after purification by enterokinase respectively.

### Construction and cloning of IFN-NS3-LAP gene in pPICZαA plasmid

The IFN-NS3-LAP gene was constructed through splicing by overlap extension PCR (SOE-PCR). The IFNα-2b gene without stop codon was amplified from plasmid pIFN using primer: RP1 (5′-AGAACAACAAACAACATCTTCCTCCTTGGATCTCAAGGACT-3′) and FP1 (5′-TGTGACTTGCCACAAACTCACTCCTTGGGTTCC-3′). The LAP gene was amplified from plasmid pLAP using primers: FP2 (5′-GAAGATGTTGTTTGTTGTTCTATGTCCTACTTGTCTACTTGTAAGACTAT-3′) and RP2 (5′-GTTCTAGATTACCTCCTATGACGGGAAGATTGCAAATGTTGGGCT-3′). The full length gene was amplified by FP1 and RP2 primers. Tag peptide sequence was introduced at 5′ end by overlap primer walk PCR (OPW-PCR) using forward primers: FP3 (5′-CACCACGGATCCGATGATGATGATAAGTGTGACTTGCCACAAACTCA-3′) and FP4 (5′-GCCTCGAGAAAAGACACCACCACCACCACCACGGATCCGATGATGAT-3′) along with RP4 primer. The restriction sites of *xbaI* and *xhoI* were introduced at 5′ and 3′ end of fusion gene by FP4 and RP2 primer respectively. The gene construct was cloned in pPICZαA plasmid digested with *xbaI* and *xhoI* restriction enzymes. The resultant plasmid was designated as pPICZαA-IFN-NS3-LAP.

### Construction and cloning of LAP-NS3-IFN gene in pPICZαA plasmid

LAP-NS3-IFN gene was constructed in the same manner as described for IFN-NS3-LAP gene. Briefly, LAP gene was amplified from LAP plasmid without its stop codon using primer RP1′ (5′-AGAACAACAAACAACATCTTCCCTCCTATGACGGGAAGATTG-3′) and FP1′ (5′-TTGTCTACTTGTAAGACTATTGATATGGAATTGGTTAAGAGAAAGAGAATTGAAG-3′). The IFNα-2b gene was amplified from pIFN plasmid using primers FP2′ (5′-GAAGATGTTGTTTGTTGTTCTATGTCCTACTGTGACTTGCCACAAACTCA-3′) and RP2′ (5′-GCCTTCTAGATTACTCCTTGGATCTCAAGGACTCTTGCAAGTTGGTAG-3′). The full length gene LAP-NS3-IFN was amplified by SOE-PCR using FP1′ and RP2′ primers. The tag peptide sequence was introduced by OPW-PCR using FP3′ (5′-CACCACGGATCCGATGATGATGATAAGTTGTCTACTTGTAAGACTATTGAT-3′) and FP4 primer along with RP2′ primer. The primers FP4 and RP2′ introduced *xbaI* and *xhoI* sites at 5′ and 3′ end of gene. The fusion gene was cloned in pPICZαA plasmid digested with *xbaI* and *xhoI* restriction enzymes. The recombinant plasmid was designated as pPICZαA-LAP-NS3-IFN.

### Transformation in Pichia pastoris and screening of transformants

*Pichia pastoris* strain GS115 cells were transformed with 3 µg of recombinant plasmids (pPICZαA-IFN-NS3-LAP and pPICZαA-LAP-NS3-IFN) linearized with *SacI* restriction enzyme using Easy Comp^TM^ kit (Invitrogen) according to the instructions of manufacturers. The transformants were selected on YPD agar (1% yeast extract, 2% peptone, 2% dextrose, 2% agar) plates augmented with 100 µg/ml of zeocin incubated at 28 °C for 3–5 days. The clonal isolates of 6–10 transformants were also checked for integration of expression cassette in *pichia* genome by colony PCR and PCR using genomic DNA of transformants with 5′AOX1 primer (5′-GACTGGTTCCAATTGACAAGC-3′) and 3′ AOX1 primer (5′-GCAAATGGCATTCTGACATCC-3′). The transformants which showing growth on YPD agar plates supplemented with zeocin up to 1.5 mg/ml–2.0 mg/ml were selected for protein expression.

### Expression of IFN-NS3-LAP and LAP-NS3-IFN proteins in Pichia pastoris

The selected Mut^+^ transformants of each pPICZαA-IFN-NS3-LAP and pPICZαA-LAP-NS3-IFN were cultivated in shake flask culture under the control of inducible alcohol oxidase promoter (AOX1). The transformants refreshed in YPD broth were inoculated in 10 ml of BMGY medium (1% yeast extract, 2% peptone, 100 mM potassium phosphate pH 6.0, 1.34% YNB, 4 × 10^−5^% biotin, 1% glycerol) in 100 ml of baffled flask at 28 °C with shaking at 300 rpm until the OD_600_ of culture reached to 2.0–6.0. The cells were harvested and resuspended in 100 ml of BMMY medium (100 mM potassium phosphate buffer pH 5.0, 1.34% YNB, 1% yeast extract, 2% peptone, 4 × 10^−5^ biotin, 0.5% methanol) at OD_600_ 1.0 of culture in 1000 ml baffled flask at 20 °C with shaking at 300 rpm for 5 days (120 hours). The cultures were induced with methanol at final concentration of 1.0% after interval of 24 hours. The supernatant were collected and extracellular proteins were concentrated 50 times by ammonium sulphate precipitation. The protein expression was analyzed by 12% SDS-PAGE^35^ under reducing and non-reducing concentrations.

### Purification of fusion proteins

The supernatant (100 ml) of induced cells expressing fusion proteins (IFN-NS3-LAP and LAP-NS3-IFN) was collected by centrifugation at 8000 rpm for 10 minutes. The collected supernatant was concentrated 50 times by ammonium sulphate precipitation at 100% saturation. The fusion proteins were solubilized in buffer A (20 mM sodium phosphate, 500 mM NaCl pH 7.8). The concentrated proteins were loaded on Ni-NTA resin (Invitrogen) pre-equilibrated with buffer A. The column was washed with buffer A and the bound protein was eluted with buffer B (20 mM sodium phosphate, 500 mM NaCl pH 6.0, 250 mM imidazole). The imidazole was removed from collected fractions using Amicon centrifugal columns (cut off- 10 kDa, Millipore). The purified proteins were concentrated and analyzed by 12% SDS-PAGE. The protein concentration was determined by Bradford assay^36^. Bovine serum albumin (BSA) was used as standard.

### Removal of Histidine Tag by enterokinase

Histidine tag from N-terminal of purified proteins was removed by incubating with the 5 units of enterokinase (Sigma Aldrich) per µg of protein in buffer (10 mM Tris-Cl pH 8.0, 10 mM CaCl_2_) at 37 °C overnight. The removal of histidine tag was confirmed by western blot using anti-histidine antibodies. The fusion protein without enterokinase treatment was used as positive control. The fusion proteins were purified using Amicon ultra 0.5 ml spin column (cut off 30 kDa, Millipore).

### Western blotting

After SDS-PAGE analysis, the fusion proteins were also characterized by western blotting. The proteins separated by SDS-PAGE were electroblotted on nitrocellulose membranes with semi-dry electroblotting apparatus (Bio-Rad). The membranes were blocked using 5% skim milk in TBST (50 mM Tris-Cl pH 7.6, 150 mM NaCl, 0.05% Tween 20). The blots were probed with both mouse anti-human IFNα2 antibodies or mouse anti-human LAP antibodies (Santa Cruz Biotechnology) as primary antibodies (1: 3000 dilution) and goat anti-mouse IgG-alkaline phosphatase conjugated (Santa Cruz Biotechnology) as secondary antibody (1:5000 dilution). The western blots were developed using alkaline phosphatase substrate (NBT/BCIP).

### *In vitro* cleavage assay of fusion proteins

The fusion proteins were incubated overnight with recombinant HCV NS3 protease of genotype 3a (produced in our lab) in 1: 10 molar ratio at 37 °C in total volume of 100 µl using 1x reaction buffer (50 mM Tris-Cl pH 7.5, 150 mM NaCl, 0.05% Tween 20, 20% glycerol, 1 mM DTT). In order to check the chances of fusion protein activation in blood during systemic delivery, the *in vitro* cleavage assay was also performed using serum and PBMCs (4 × 10^7^ cells/assay) isolated from HCV infected patients (with different viral loads and stages of liver fibrosis) instead of recombinant NS3 protease in assay. The cleavage of fusion proteins was analyzed by 12% SDS-PAGE, western blot and by sandwich ELISA. For sandwich ELISA, 100 µl of cleavage reaction mixture was added to wells of 96 well microtitre plate pre-coated with mouse anti-human LAP antibody (10 µg/ml diluted in 0.5 M carbonate buffer pH 9.5) and incubated for 4 hours at room temperature. The wells were washed thrice with 1x PBS and incubated with 100 µl of mouse anti-human IFNα2 antibody (2.5 µg/ml) for 4 hours at room temperature. The immunocomplex was detected using 100 µl of alkaline phosphatase conjugated goat anti-mouse IgG (1: 5000 dilution). After washing with 1x PBS, the color reaction was developed using 100 µl of p-nitrophenyl phosphate (PNPP) by incubation of 10 minutes at 37 °C. The absorbance was recorded at 405 nm. The cleavage was monitored using standard curve developed with different concentrations of intact fusion protein by aforementioned sandwich ELISA. The negative control reaction without NS3 protease was also performed.

### Cytopathic effect inhibition assay

The *in vitro* biological activity of both fusion proteins in latent form (without treatment of HCV NS3 protease), and activated form (after treatment with HCV NS3 protease) was determined by inhibition of cytopathic effect in 549 cells (human lung carcinoma cell line) infected with encephlomyocarditis virus (EMCV). The cells were seeded in 96 well culture plate (4.5 × 10^5^ cells/well) and grown to 80% confluency at 37 °C with 5% CO_2_ in DMEM medium containing 10% fetal bovine serum (FBS), 100 U/ml of penicillin and 100 µg/ml streptomycin. The cells were exposed to the serial dilutions of fusion proteins (latent and activated forms) and IFNα2b standard (PBL Biomedical Laboratories, Piscataway, NJ) and incubated for 24 hours. For real time activation, NS3 protease was also added along with latent fusion proteins in 1: 10 ratio. To each well EMC virus was added at optimal concentration and incubated for addition 24 hours. The viability of cells was determined by staining with 1% crystal violet. After removal of excessive dye and washing of cells, the dye in cells was solubilized using 70% ethanol. The absorbance was recorded at 580 nm in ELISA microplate reader. The experiment was performed in duplicate. The antiviral activity was expressed as EC_50_ (concentration that protected 50% of cells) and determined using GraphPad Prism software. The cells treated with IFNα2b standard were considered as positive control and cells without any protein exposure were considered as negative control. The U/ml and specific activity (U/mg) of fusion proteins was calculated with reference to standard IFNα2b.

### Anti-HCV activity

The anti-HCV activity of fusion proteins (latent and activated forms) was also assessed. The *in vitro* replication of HCV was established in Huh7 cells as described by El-Awady *et al*.^37^ with slight modifications. Briefly, the cells (3 × 10^5^ cells/ well) were cultured in 3 ml of DMEM medium containing 10% FBS along with 100 U/ml of penicillin and 100 µg/ml streptomycin at 37 °C in 5% CO_2_ for 48 hours to semi-confluency. After washing with FBS free DMEM medium, the cells were inoculated with 500 µl of serum of HCV genotype 3a patient with high viral titer (>1 × 10^8^ IU/ml) and 500 µl of FBS free DMEM medium. After incubation of 90 minutes at 37 °C in 5% CO_2_, 2 ml of DMEM medium was added containing FBS to make its overall concentration to 10%. The cells were incubated for 48 hours at 37 °C in 5% CO_2_. The cells were re-seeded in 6 well culture plate and exposed to different concentrations of fusion proteins (0.01 ng/ml-100 ng/ml) in latent form (without treatment of HCV NS3 protease), activated form (after treatment with HCV NS3 protease) and with unconjugated IFNα2b as control (PBL Biomedical Laboratories, Piscataway, NJ). The NS3 protease was added in to the wells with latent fusion proteins in 1: 10 molar ratio for real time activation. The cells were incubated for 24 hours at 37 °C in 5% CO_2_. At the end of assay, RNA was isolated from cells using GeneJET RNA isolation kit (ThermoScientific) following the instructions of manufacturers. The HCV RNA was quantified using real time HCV quantification kit “Real ART^TM^ HCV RG RT PCR kit” (Corbett Research Qaigen, Germany) following the instructions of manufacturers. The anti-HCV activity of fusion proteins was determined relative to the unconjugated IFNα2b. All the experiments were performed in triplicate.

## Discussion

In modern medicine, fight against the viral infections is considered as most challenging area. Vaccination is generally used to cure the viral infection. However, for some viruses which cause worldwide health problems like human immunodeficiency virus (HIV) and hepatitis C virus (HCV), vaccines have not been developed yet. Therefore, over the past decade, efforts have been made to find and/or develop the potent anti-viral agent.

HCV virus annually infects over 170 million people worldwide^38^. Initially, interferon therapy was considered as standard of care for HCV infection. In systemic route of delivery, interferon triggers antiviral effect not only in liver but also activates blood leukocytes that results in adverse effects of therapy^28^. Due to the poor pharmacokinetics (short serum half-life, bioavailability) and pharmacodynamics (adverse effects and toxicity), interferon therapy is not preferred now^39,40^. The approach of targeting therapeutics to specific cells or tissues is used to improve the safety and efficacy of therapeutic. To improve interferon therapy, interferon alpha has been targeted to liver via domain antibody specific to hepatocyte restricted antigen, asialoglycoprotein receptor^28^ and by fusion of galactosyl human serum albumin that is targeted to asialoglycoprotein receptor on hepatocytes^29^. Current trends to treat HCV infection include, use of novel molecules named as Direct acting Antivirals (DAAs) that target to viral proteins i-e. HCV non-structural proteins (NS3 protease, NS5B polymerase and NS5A) which are considered as ideal drug targets^41^. However, success of DAA therapy is challenged by ability of virus to overcome the selective drug pressure and develop escape mutants that resist action of antiviral drugs^42^. As a result, patients show resistance to DAA and left with no choice except interferon therapy.

On the other side, there is another approach of taking advantage of specific viral activity instead of targeting them. In this approach, antiviral agent is made inactive by fusion partner linked via viral protease cleavage site. The antiviral agent can be released only by removal of fusion partner by the action of viral proteases at the site inserted during fusion and results in selective eradication of virus infected cells^43^. This concept is firstly introduced in the form of sitoxin by Varshavsky^44^. In later studies, viral protease activating antiviral agent “zymogens” were developed by circular permutation of bovine RNase A and activated by plasmodium falciparum, HIV and HCV proteases^31,32,45^, activation of MazE-MazF antitoxin- toxin fusion protein by either HIV protease, HCV NS3 protease or factor Xa having specific protease cleavage site^46^.

In the present study, we developed latent IFNα2b by fusion of latency associated protein domain of human TGFβ at either its N or C-terminus through HCV NS3 protease cleavage site as liker. The LAP domain of TGFβ conferred latency to the IFNα2b by providing the protective shell. The latency cause steric hindrance and inhibits the binding of IFNα2b with its receptors. This latency can be abrogated by treating the fusion protein with HCV NS3 protease.

The genes were fused through SOE-PCR (Fig. 2). The fusion genes were expressed in *pichia pastoris* Mut^+^ strain GS115 under AOX1 inducible promoter. Under optimized conditions, the fusion proteins were expressed and purified by Histidine tag. The His-tag was removed by enterokinase enzyme. The proteins were purified by one step molecular weight cut off spin column (MCO of 30 kDa.). The fusion proteins were produced as homodimer as evident from the western blot and SDS-PAGE gel under non-reducing and reducing conditions (Fig. 6A,B). The formation of disulfide bond in LAP domains is required for shell structure which attributes latency to the fusion protein^30^. In a number of different studies, LAP domain of TGFβ has been fused to the cytokines to make them latent until reached at disease site and activated by cleavage off LAP domain by enzymes present at pathological site. Vessillier *et al*.^47^ reported fusion of mouse interferon β with LAP domain of TGFβ via linker of matrix metalloproteinase (MMP) cleavage site. Mullen *et al*.^48^ reported many latent cytokines IL-1ra, IL-10, IL-4, IL-17 by fusion of LAP domain of TGFβ via MMP cleavage site as linker. Vessillier *et al*.^49^ also reported fusion of a number of anti-inlflammatory peptides (vasoactive intestinal peptide, α-melanocyte-stimulating hormone and γ3MSH) to LAP via cleavable MMP linker.

To activate the latent fusion proteins (IFNα2b-NS3-LAP and LAP-NS3-IFNα2b) developed in the present study, the proteins were *in vitro* treated with HCV NS3 protease. The protease treatment resulted in formation of two fragments corresponding to the size of LAP (28.5 kDa) and IFNα2b (18.1 kDa) (Fig. 7A). The cleavage was further confirmed by western blot (Fig. 7B) using mouse antihuman LAP antibodies and mouse antihuman IFNα2 antibodies. Subsequently, the biological activity of latent and activated IFNα2b fusion proteins was assessed.

In most of the aforementioned studies, the latent proteins were developed by fusion of LAP domain via MMP cleavable linker. Here, we used HCV NS3 protease cleavable linker to make IFNα2b latent by fusion of LAP. The NS3 protease is a non-structural protein of HCV and required for fragmentation of HCV polypeptide in different proteins. The NS3 protease is present in the cells whereas receptors of IFNα2b are present on cell surface. In the present study, we hypothesized the activation of latent IFNα2b at target site by extracellular HCV NS3 protease. As evidences are present for presence of extracellular HCV NS3 protease. The presence of NS3 protease on the surface of HCV infected cells by immunostaining has been reported by Sakata *et al*.^34^. They reported that a high concentration (50 µg/ml) of HCV NS3 protease could be present on surface of HCV infected cells in close proximity of TGF-β receptor. The exact mechanism by which NS3 protease comes out of the cells is not known. One possibility is the passive leakage from injured hepatocytes likewise the alanine aminotransferase and aspartate aminotransferase. The other possibility is the secretion of HCV NS3 protease from HCV infected cells through golgi complex as the non-structural protein 1 (NS1) of dengue virus and west nile virus are secreted from dengue and west nile virus infected cells. As all these viruses belong to the same family “Flaviviridae”^50^. Zhang *et al*.^51^ also reported the presence of cell binding sequence “Arg-Gly-Asp” in close proximity to the major linear antigenic region of HCV NS3 protease. The antibodies against HCV NS3 protease in serum of chronic hepatitis C patients also indicate the presence of extracellular NS3 protease^51,52^. However, there is not a single report about the presence of free circulating NS3 protease in serum of chronic hepatitis C patients.

Therefore in the present study, the level of HCV NS3 protease was determined in the serum of CHC patients. The results showed that NS3 was not detected in the serum of HCV patients. It may be due to the low level of free NS3 protease circulating in serum. There is also the possibility that NS3 is present in the form of immunocomplex in serum that is why remained undetectable. Although dengue virus NS1 has been detected in the serum of dengue virus infected patients at high level (50 µg/ml) but only in acute phase of disease^53^.

Although, liver is the main site for HCV replication but it is not strictly hepatotropic. The extrahepatic replication sites of HCV, mainly, peripheral blood mononuclear cells (PBMCs) which includes lymphocytes (T cells, B cells and natural killer cells) and monocytes, have also been reported^54^. In present study, *in vitro* cleavage assay of intact latent IFNα2b was also performed using PBMCs (as expected source of NS3 protease) isolated from CHC patients to investigate the possibility of intact latent IFNα2b activation by PBMCs. The cleavage of intact fusion protein was not observed by PBMCs. It may be due to the absence of NS3 protease on the surface of PBMCs. Therefore, there are less likely chances of latent IFNα2b developed in the present study to be activated in the serum of CHC patients until reached at the target site (liver) and activated by NS3 protease present at the surface of HCV infected cells.

The biological activity of latent and activated IFNα2b fusion proteins was assessed by cytopathic effect inhibition assay on A594 cells infected with EMC virus. The results of the study revealed that latent form has 0.2% to 0.16% while pre-activated fusion proteins had 36%-34% of antiviral activity in comparison to the standard unconjugated IFNα2b. The biological activity was further reduced to ~4–5% for real time activated fusion proteins. In the case of anti-HCV activity of latent and activated IFNα2b fusion proteins, the results indicating that the latent forms were activated by the extracellular HCV NS3 protease released from HCV replicating cells and present on the surface of cells. The results of the study showed that antiviral activity of latent chimeric proteins was comparable to the activated chimeric protein up to tested concentration of 0.5 ng/ml. The viral titre was reduced to 25% approximately at this concentration. Further increase in concentration of latent proteins did not show any significant reduction in viral titre. It may be due to the exhaustion of NS3 protease present on HCV replicating cells. The 50% reduction in HCV viral load was recorded at 0.4 ng/ml and 1.0 ng/ml by unconjugated IFNα2b and pre-activated form of IFNα (same on molar basis). The results should that HCV NS3 protease is present on the surface of HCV replicating cells in amount that activated half of the effective concentration of IFNα2b fusion protein.

The latent forms of IFNα2b also showed some antiviral activity in non-HCV replicating cells which indicating that chimeric proteins were not fully latent. However, LAP-NS3-IFN exhibited less biological activity than IFN-NS3-LAP (Table 2) which showed that LAP confers better latency in homodimeric form when fused to the N-terminal of the IFNα2b as found in native TGFβ. Therefore, it is clear that fusion of LAP at N-terminus conferred better latency that fusion at C-terminus of IFNα2b. Fusion of LAP at N-terminus of protein provides better protective shell as found in native TGF-β.

The findings of the study supported the hypothesis of this study of activation of latent chimeric proteins (latent IFNα2b) at site of HCV infection (HCV infected cells) by NS3 protease released from cells.

## Conclusion

The fusion of IFNα2b to LAP overwhelmed the limitation of side effects of interferon therapy. The IFNα2b fusion protein delivered by systemic route of delivery will not bind with off target cellular receptors until reach at target site (liver) and activated by NS3 protease present on the surface of HCV replicating cells. However the safety profile will be improved at the cost of the half of the activity. Therefore, IFNα2b, modified as latent cytokine can an option for treatment of special population of HCV patients in combination with direct anting antivirals rather than alone with improved safety profile. However, further pharmacological assessments in animal models are required to determine the safety and efficacy of latent IFNα2b in the context of whole organism.

## Supplementary information

Supplementary Information
