# Supplementary material for: Development of latent Interferon alpha 2b as a safe therapeutic for treatment of Hepatitis C virus infection
Source: Sci Rep. 2019 Jul 26;9:10867. doi: 10.1038/s41598-019-47074-y (PMC6659634; doi:10.1038/s41598-019-47074-y)
Supplement: Supplementary file 1 — Supplementary Information [file 41598_2019_47074_MOESM1_ESM.pdf]

1a) Title

Development of latent Interferon alpha 2b as a safe therapeutic for treatment of Hepatitis C virus infection

1b) Authors

Iram Gull*[1], Muhammad Shahbaz Aslam,*[1] Imran Tipu*[1], Roohi Mushtaq*[1], Tehseen Zamir Ali*[2] and Muhammad Amin Athar*[1]

1c) Place of work

*[1] Institute of Biochemistry and Biotechnology, Quaid-i-Azam Campus, University of the Punjab, Lahore, 54590, Pakistan.

*[2] Punjab University Health Centre, Quaid-i-Azam Campus, University of the Punjab, Lahore, 54590, Pakistan.

1d) E-mail Addresses of authors

Iram Gull-------------------------------------iram.ibb@pu.edu.pk
Muhammad Shahbaz Aslam--------------shahbaz.ibb@pu.edu.pk
Imran tipu------------------------------------imran.tipu1@gmail.com
Roohi Mushtaq------------------------------roohimushtaq@gmail.com
Tehseen Zamir Ali--------------------------twinklestar_47@yahoo.com
Muhammad Amin Athar------------------amin.ibb@pu.edu.pk

1e) Address for Correspondence,

Iram Gull
Institute of Biochemistry and Biotechnology, Quaid-i-Azam Campus
University of the Punjab, Lahore, 54590, Pakistan.
Email: iram.ibb@pu.edu.pk
Fax: 92-42-99230242
Telephone: 92-42-99230134

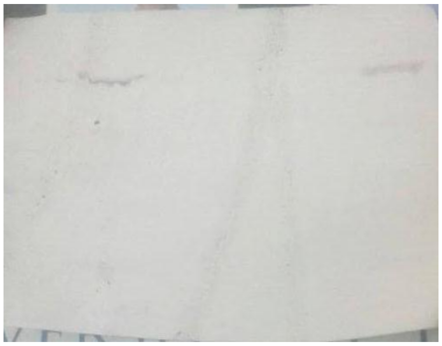

Figure 1. Western blotting of purified Homodimers of IFNα2b-NS3-LAP and LAP-NS3-IFNα2b detected by mouse anti-human IFNα2 antibodies

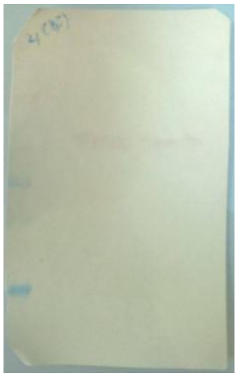

Figure 2. IFNα2b-NS3-LAP and LAP-NS3-IFNα2b detected with mouse anti-human IFNα2 antibodies by western blot under reducing conditions.

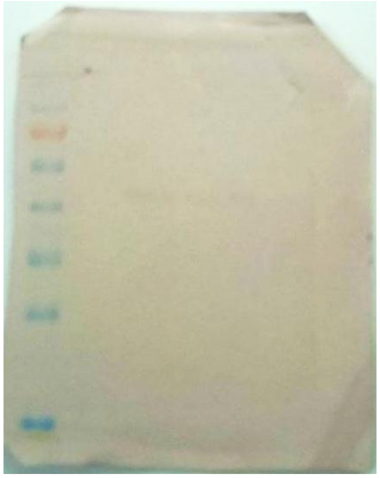

Figure 3. IFNα2b-NS3-LAP and LAP-NS3-IFNα2b detected with mouse anti-human LAP antibodies by western blot under reducing conditions.

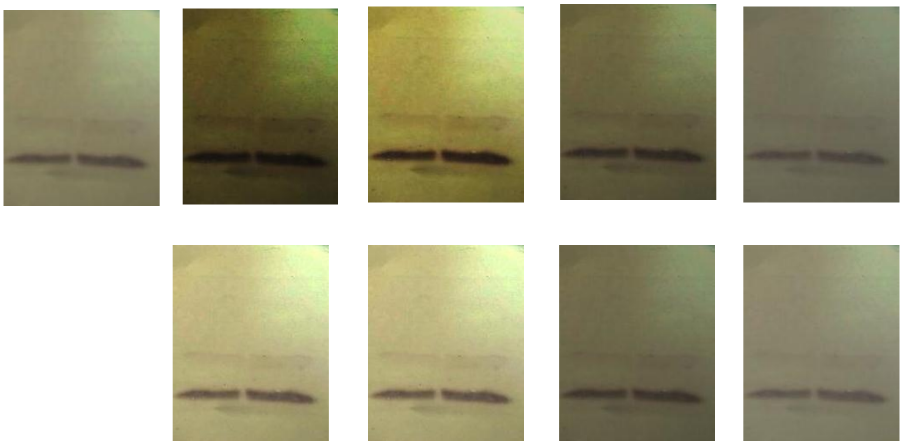

Figure 4. Western blot analysis of IFNα2b and LAP fragment produced after cleavage of fusion proteins IFNα2b-NS3-LAP (Lane 1) and LAP-NS3-IFNα2b (Lane 2) by HCV NS3 protease using mouse anti-human IFNα2 antibodies and mouse anti-LAP antibodies as primary antibodies.(blot with multiple exposures).
